# Identification of vitamin D sensitive pathways during lung development

**DOI:** 10.1186/s12931-016-0362-3

**Published:** 2016-04-27

**Authors:** Ling Chen, Richard Wilson, Ellen Bennett, Graeme R. Zosky

**Affiliations:** School of Medicine, Faculty of Health, University of Tasmania, Hobart, Tasmania Australia; Central Science Laboratory, University of Tasmania, Hobart, Tasmania Australia

**Keywords:** Vitamin D deficiency, Proteomic mass spectrometry, Pulmonary surfactant-associated protein B, Collagen type Ι alpha 1, Lung development

## Abstract

**Background:**

We have previously shown that vitamin D deficiency has a detrimental impact on lung development. In this study, we aimed to identify the mechanisms linking vitamin D with lung development using a mouse model of dietary manipulation.

**Methods:**

Female offspring were euthanized at different time-points; embryonic day (E)14.5, E17.5 or postnatal day (P)7. Lung tissue was collected for mass spectrometry-based proteomic analysis. Label-free quantitation was used to identify the differentially expressed proteins and ELISA confirmed the expression of selected proteins. Lungs from separate groups of mice were fixed and processed for stereological assessment of lung structure.

**Results:**

No differences in protein expression between vitamin D deficient and replete mice were detected at E14.5 and E17.5, whereas 66 proteins were differentially expressed in P7 lungs. The expression of pulmonary surfactant-associated protein B (SP-B) and peroxiredoxin 5 (PRDX5) were reduced in P7 lungs of vitamin D deficient mice, while the production of collagen type Ι alpha 1 (COL1A1) was higher in lungs of vitamin D deficient mice. There were no differences in lung volume, parenchymal volume, volume of airspaces or surface area of airspaces between vitamin D deficient and vitamin D replete mice across three time-points.

**Conclusions:**

The difference in protein expression during the early postnatal time-point suggests that vitamin D deficiency may induce alterations of lung structure and function in later life during alveolarization stage through impaired pulmonary surfactant production and anti-oxidative stress ability as well as enhanced collagen synthesis. These data provided a plausible mechanism linking maternal vitamin D deficiency with altered postnatal lung function.

## Background

Vitamin D has roles in both skeletal and non-skeletal health, and vitamin D deficiency is recognized as a prevalent health problem [1, 2]. A plethora of studies have shown cross-sectional associations between vitamin D deficiency and chronic respiratory diseases such as asthma, chronic obstructive pulmonary disease, and cystic fibrosis [3–5]. However, these associations are confounded by the effect of chronic disease on physical activity levels [6] which are highly correlated with sun exposure and, therefore, vitamin D synthesis [7]. In line with this, while studies in animal models have suggested vitamin D supplementation may ameliorate markers of chronic lung disease [8, 9], clinical trials using vitamin D supplementation have been disappointing [10, 11].

While clinical studies have shown no benefit of vitamin D supplementation in established disease, other studies have suggested that vitamin D deficiency may be a precursor to the development of respiratory disease [12]. For example, maternal vitamin D deficiency is associated with impairments in postnatal lung function [13, 14] and an increased risk of developing asthma [15, 16]. In support of this, we have recently demonstrated that *in utero* vitamin D deficiency is sufficient to cause deficits in lung structure and function using a mouse model [17, 18]. Genomic analyses of lung tissue from mice and humans have identified a range of genes that are involved in lung development [19] that contain vitamin D response elements (VDREs) [20, 21] suggesting a wide range of roles for vitamin D in normal lung growth. In addition, in vitro and in vivo studies have suggested that vitamin D may be involved in epithelial-mesenchymal interactions [22, 23], calcium regulation in alveolar type II cells [24] and surfactant metabolism [25] during lung maturation. Taken together, these observations suggest that vitamin D is critical during lung development; however, no study has assessed the pathways that are directly altered by vitamin D deficiency during fetal lung development in vivo.

The aim of this study was to identify lung development pathways that are sensitive to vitamin D deficiency. Specifically, using an established mouse model of vitamin D deficiency, we aimed to determine if maternal vitamin D deficiency 1) has an influence on the protein expression in embryonic and neonatal lungs and 2) results in alterations in lung morphology during early lung development.

## Methods

### Mouse model

A mouse model of vitamin D deficiency was established by dietary manipulation as described previously [17]. Briefly, three weeks old female BALB/c mice (ARC, Murdoch, Western Australia) were fed with vitamin D-deficient (0 vitamin D_3_) or –replete (2195 IU/kg vitamin D_3_) diets (Specialty Feeds, Glen Forrest, Western Australia) for at least 5 weeks before mating with replete male mice. The caloric content of these two diets were similar (deficient, 15.3 MJ/kg; replete 15.8 MJ/kg), and the deficient diets were supplemented with calcium 25 g/kg to avoid hypocalcaemia. Mice were housed in a room with a 12:12 h ultraviolet B-free light/dark cycle. At key developmental time-points embryonic day (E)14.5, E17.5 or postnatal day (P)7, mice were euthanized by overdose with ketamine: xylazine (800 mg/kg: 40 mg/kg). Lungs of E14.5, E17.5 and P7 vitamin D deficient and replete mice were snap frozen in liquid nitrogen for proteomic analysis or immersion fixed in formalin for histological assessment. We focussed on female mice for these experiments based on our previous study which showed the female lung is most susceptible to the effects of early life vitamin D deficiency [17]. To ensure independence of the analysis each mouse was from a different litter, the sex of the pups sex was determined by PCR [26]. Following euthanasia, serum from pregnant dams was collected by cardiac puncture for assessment of maternal serum 25-hydroxyvitamin D [25(OH)D] levels using a 25-hydroxyvitammin D EIA kit (Immunodiagnostic Systems Limited, Boldon, UK) according to the manufacturer’s instruction. All studies were performed according to the National Health and Medical Research Council (NHMRC) guidelines and were approved by The University of Tasmania Animal Ethics Committee.

### Protein sample preparation

Lungs were homogenized and proteins were extracted and digested using trypsin according to established methods [27]. Briefly, the whole lung was homogenized in denaturation buffer (7 M urea, 2 M thiourea, 30 mM Tris, pH8.0) with EDTA-Free protease inhibitor cocktail (Roche, Basel, Swizerland), and rotated for 2 h at 4 °C. Protein extracts were precipitated with 9 volumes of ethanol overnight at −20 °C, and protein pellets were air-dried and resuspended in 200 μl solubilisation buffer (7 M urea, 2 M thiourea, 30 mM Tris, pH8.0). Protein concentrations were estimated by Bradford assay and stored at −80 °C for future use. Protein samples for liquid chromatography tandem mass spectrometry (LC-MS/MS) analysis were sequentially incubated in 10 mM dithiothreitol (DTT) overnight at 4 °C and then 50 mM iodoacetamide incubation for 2 h at 25 °C in the dark. Proteins were co-precipitated with 1 μg of trypsin overnight at −20 °C in 1 ml of methanol. The trypsin-protein samples were air-dried and reconstituted in 100 mM ammonium bicarbonate followed by trypsinization at 37 °C for 5 h with addition of 1 μg of trypsin after 2 h. Digests were terminated by freezing on dry ice.

### Orbitrap mass spectrometry

Protein expression was analysed by nano LC-MS/MS using a Surveyor HPLC system in line with a LTQ-Orbitrap XL controlled using XCalibur 2.0 software (Thermo Fisher Scientific, Waltham, MA, USA). Protocols were based on previously described conditions for peptide separation and data-dependent MS/MS [28]. The LTQ-Orbitrap XL was controlled using XCalibur 2.0 (Thermo Fisher Scientific, MA, USA) and operated in data-dependent acquisition mode where survey scans (m/z 460–2000) were acquired in the Orbitrap at a resolving power of 60,000. MS/MS spectra were concurrently acquired in the LTQ mass analyser on the eight most intense ions from the FT survey scan. Unassigned and singly charged precursor ions were not selected for fragmentation and 30-s dynamic exclusion (repeat count 1 exclusion list size 500) was used. Fragmentation conditions in the LTQ were: 35 % normalized collision energy, activation q of 0.25, 30 ms activation time and minimum ion selection intensity of 3000 counts.

### Protein identification and bioinformatics analysis

The LTQ-Orbitrap RAW data files were imported into MaxQuant software (version 1.5.1.2; http://www.maxquant.org/). The extracted MS/MS spectra were searched against the complete Mus musculus reference proteome (ID 000000589; updated on 02/10/2014) comprising 44,455 protein entries using the Andromeda search engine. Default group-specific and global parameters for protein identification were used including specific digestion by trypsin allowing a maximum of two missed cleavages, variable oxidation of methionine and acetylation of protein amino termini, and fixed carbamidomethylation of cysteine. The tolerances for first peptide searches were set to 20 ppm and reduced to 4.5 ppm for the main searches. A false discovery rate (FDR) of 0.01 was used for peptide-spectrum matches and protein identification. Normalized label-free quantitation (LFQ) values were imported from the MaxQuant ProteinGroups output file for statistical analysis in the Perseus software package (version 1.5.2.6; http://perseus-framework.org/). Z-score normalized data were analysed by principal component analysis (PCA) to determine if any intrinsic clustering or outliers existed within the data set. Subsequently, two approaches were used for identification of differential expression of proteins; 1) based on the subset of proteins detected in all samples or, 2) based on proteins detected in a minimum of three biological replicates in one or more treatment groups where missing LFQ values were statistically imputed (based on the distribution of LFQ intensity values). The differential expression of an individual protein between vitamin D deficient and replete groups was evaluated by t-tests using FDR adjusted *p* values.

### Enzyme-linked immunosorbent assay (ELISA)

A subset of proteins with biologically plausible links to altered postnatal lung function was selected for independent confirmation of expression in lungs to the proteomic assays using ELISA. Specifically, the production of pulmonary surfactant-associated protein B (SP-B) (CUSABIO, Wuhan, China), collagen type Ι alpha 1 (COL1A1) (CUSABIO), and peroxiredoxin 6 (PRDX6) (Abcam, Cambridge, United Kingdom) in tissue homogenates was measured according to the manufacturer’s instructions. 40 μg of the soluble protein extract from each lung was loaded into the ELISA. The absorbance was read at 450 nm or 570 nm using a spectrophotometer (Spectramax M2; Molecular Devices, Sunnyvale, CA, USA).

### Western blotting assay

The expression of peroxiredoxin 5 (PRDX5) (Abcam) and myosin-11 (MYH11) (Abcam) in lung homogenates was assessed using western blotting assay. 20 μg of the soluble protein extract from each lung was denatured by heating the protein at 70 °C for 10 min and size fractionated on Bolt 4–12 % Bis-Tris Plus Gel (Thermo Fisher Scientific). The protein bands were transferred to polyvinylidene fluoride (PVDF) using iBlot Dry Blotting System (Thermo Fisher Scientific). Automate immunodetection was performed using iBand Western System (Thermo Fisher Scientific) with 0.587 μg/mL of rabbit anti-PRDX5 antibody (Abcam) or 2 μg/mL of rabbit anti-MYH11 antiboy (Abcam) followed by 0.25 μg/mL of goat anti-rabbit Ig-HRP (Dako, Glostrup Municipality, Denmark). Chemiluminescent detection was performed using Immobilon Western Chemiluminescent HRP Substrate (Millipore, Billerica, MA, USA) and bands were analysed using Kodak Image Station 4000MM and Carestream Molecular Imaging Software (Carestream Health, Rochester, NY, USA). The amount of protein present in each sample was determined as the densitometric density. Glyceraldehyde 3-phophate dehydrogenase (GAPDH) was used as a loading control, and the amount of PRDX5 or MYH11 was normalized to GAPDH, and the results were expressed as relative density to the standard.

### Lung stereology

Lung structure was assessed according to ATS/ERS guidelines for the quantitative assessment of lung structure [29] The lungs were immersed in 10 % formalin for 24 h prior to embedding in paraffin at a random orientation according to IUR principles [30]. The whole lung was serially sectioned at regular intervals (75 μm, 300 μm, and 400 μm for E14.5, E17.5, and P7 respectively) at a constant thickness (5 μm), with a random starting point between 0 and the interval thickness. Sections were stained with haematoxylin-eosin for the assessment of lung structure. Multistage stratified sampling was used to estimate morphometric parameters [31], point counting was used to obtain total lung volume (V_L_; using the Cavalieri method [32]), parenchymal volume (Vp) and volume of the airspace in the parenchyma (Va). Line and intersection counting was used to obtain the surface area of airspaces (Sa).

#### Statistics

Between-group comparisons were made using *t*-test or analysis of variance (ANOVA) with Holm-Sidak posthoc tests. Data were log-transformed where necessary to satisfy the assumptions of the test. Data were analysed in SigmaPlot (Systat, Erkrath, Germany) and reported as mean (SD). *P* values of < 0.05 were considered statistically significant.

## Results

### Maternal vitamin D levels

**Fig. 1:**
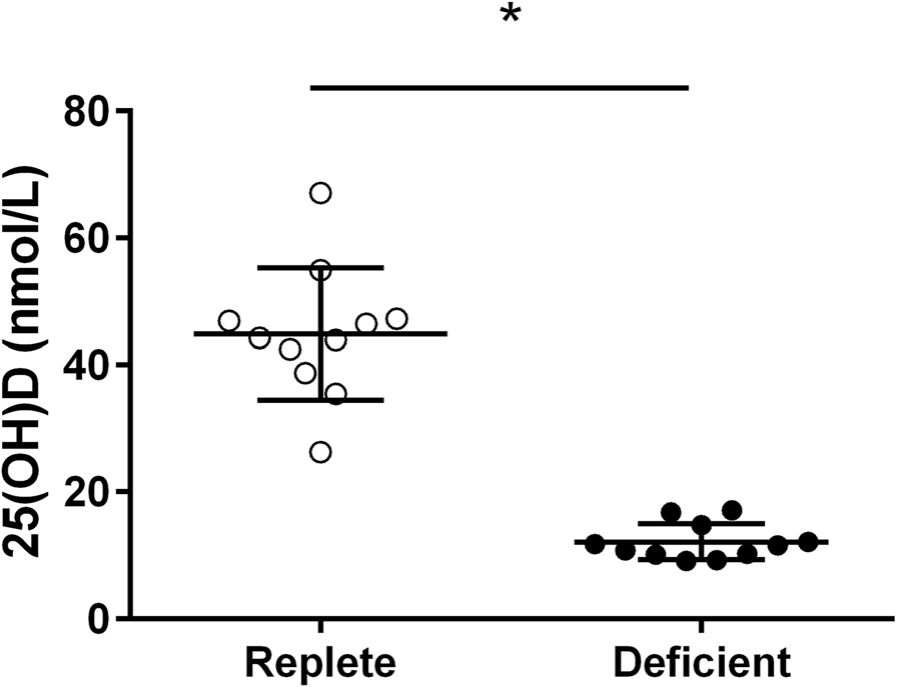
Maternal vitamin D levels. Serum 25(OH)D levels from vitamin D replete (*white circles*) and deficient (*black circles*) dams. Data are represented as mean (SD), * indicates *p* < 0.05, *n* = 11/group

Pregnant dams on the vitamin D deficient diet (*n* = 11) had significantly lower serum 25(OH)D levels than the dams on the vitamin D replete diet (*n* = 11) (*p* < 0.001) (Fig. 1).

### Principal components analysis (PCA)

**Fig. 2:**
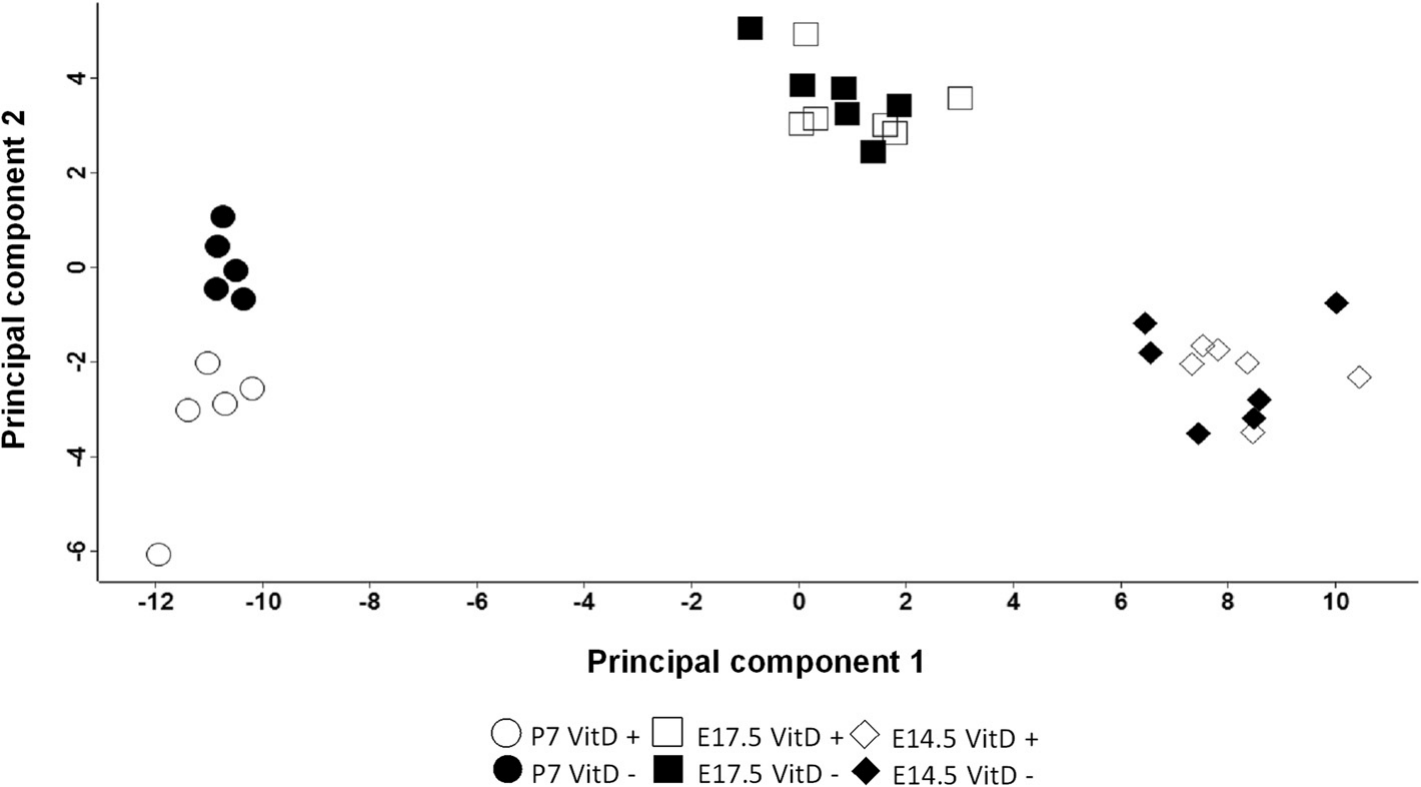
Principal components analysis map. Scatter plots of principal component 1 (x-axis) and 2 (y-axis) based on the LFQ values from the LC-MS/MS for vitamin D replete (*white symbols*) and vitamin D deficient (*black symbols*) mice at E14.5 (*diamonds*, *n* = 6/group), E17.5 (*squares*, *n* = 6/group) and P7 (*circles*, *n* = 5/group)

Whole lung protein extracts of vitamin D replete and deficient lungs (E14.5, *n* = 6/group; E17.5, *n* = 6/group; P7, *n* = 5/group) were used to perform protein mass spectrometry. In total, 1160 proteins were detected in the lungs on the basis of one or more MS/MS spectra matching to mouse peptide sequences. 240 proteins were expressed in all the samples after data normalization. PCA mapping of the first two principal components, based on LFQ values, from lungs of E14.5, E17.5 and P7 time-points showed distinct clustering based on age and vitamin D status (Fig. 2). At embryonic stages E14.5 or E17.5, the profiles of protein expression from vitamin D deficient and replete groups overlapped while the vitamin D deficient and replete mice separated into two distinct clusters at P7.

### Differential protein expression

**Fig. 3:**
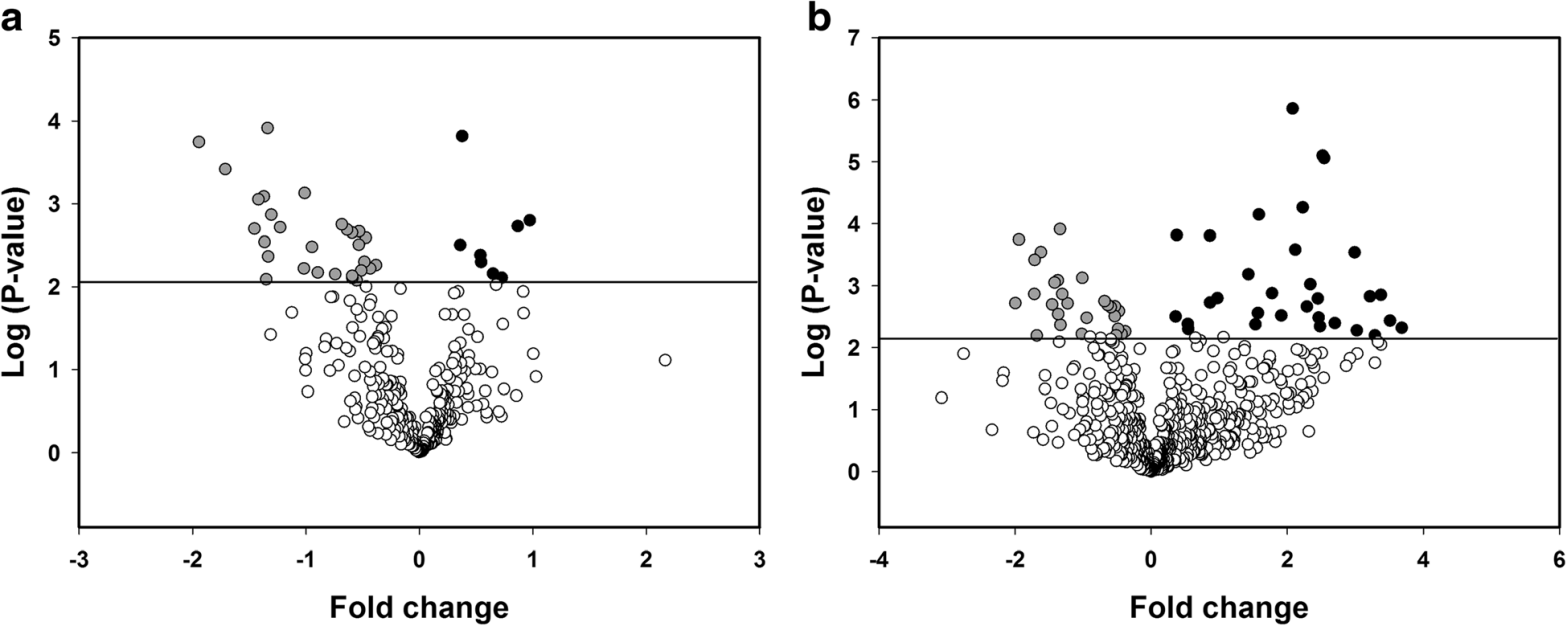
Volcano plots of differentially expressed proteins. Within the proteins that were detected in all P7 samples (**a**), there were 8 proteins with increased expression (*black circles*) and 29 proteins with decreased expression (*grey circles*) in vitamin D deficient lungs compared to replete lungs (*n* = 5/group). Within the proteins that were detected in a minimum of three P7 samples in one or more groups (**b**), there were 31 proteins with increased expression (*black circles*) and 27 proteins with decreased expression (*grey circles*) in vitamin D deficient lungs compared to replete lungs (*n* = 5/group)

In line with the PCA, no significant differences in protein expression between lungs of vitamin D deficient and replete mice were detected at E14.5 or E17.5 (*n* = 6/group). In contrast, 66 proteins were differentially expressed in P7 lungs of vitamin D deficient mice (*n* = 5) compared to vitamin D replete mice (*n* = 5) (FDR adjusted *p* < 0.05) (Table 1). Based on proteins that were detected in all samples, there were 8 proteins with increased expression and 29 proteins with decreased expression in vitamin D deficient P7 lungs compared to vitamin D replete lungs (Fig. 3a). Based on imputed LFQ values where proteins detected in a minimum of three samples in one or more groups, there were 31 proteins with increased expression and 27 proteins with decreased expression in vitamin D deficient P7 lungs compared to vitamin D replete lungs (Fig. 3b).

**Table 1:** Lists of differentially expressed proteins

Proteins that were detected in all of the P7 samples			Proteins that were detected in at least three P7 samples		
Fold change	Protein names	Gene names	Fold change	Protein names	Gene names
0.97	Lamin-A/C	Lmna	3.68	Acidic leucine-rich nuclear phosphoprotein 32 family member B	Anp32b
0.87	Phosphate carrier protein	Slc25a3	3.51	Basal cell adhesion molecule	Bcam
0.73	Myosin-11	Myh11	3.38	60S ribosomal protein L9	Rpl9
0.65	Sodium/potassium-transporting ATPase subunit alpha-1	Atp1a1	3.29	Trifunctional enzyme subunit beta	Hadhb
0.55	Myosin-14	Myh14	3.21	60S ribosomal protein L5	Rpl5
0.54	Ras-related C3 botulinum toxin substrate 1/3	Rac1/3	3.02	60S ribosomal protein L4	Rpl4
0.38	Myosin-9	Myh9	2.99	rRNA 2-O-methyltransferase fibrillarin	Fbl
0.36	Complement C3	C3	2.70	T-complex protein 1 subunit alpha	Tcp1
−0.38	Poly(rC)-binding protein 2	Pcbp2	2.54	Alpha 1 type Ι collagen	Col1a1
−0.43	Transketolase	Tkt	2.52	Small nuclear ribonucleoprotein Sm D1	Snrpd1
−0.47	Transgelin-2	Tagln2	2.48	26S protease regulatory subunit 4	Psmc1
−0.49	Glutathione S-transferase P 1/2	Gstp1/2	2.46	40S ribosomal protein S16	Rps16
−0.51	Chloride intracellular channel protein 1	Clic1	2.45	60S ribosomal protein L21	Rpl21
−0.53	26S proteasome non-ATPase regulatory subunit 13	Psmd13	2.34	Heterogeneous nuclear ribonucleoprotein H2	Hnrnph2
−0.53	Peroxiredoxin-1	Prdx1	2.23	Sarcoplasmic/endoplasmic reticulum calcium ATPase 2	Atp2a2
−0.55	Prohibitin	Phb	2.23	Interleukin enhancer-binding factor 3	Ilf3
−0.59	3-hydroxyacyl-CoA dehydrogenase type-2	Hsd17b10	2.12	Reticulon-4	Rtn4
−0.59	Protein Niban	Fam129a	2.08	Serum paraoxonase/lactonase 3	Pon3
−0.59	Tropomyosin alpha-4 chain	Tpm4	1.91	E3 ubiquitin-protein ligase NEDD4	Nedd4
−0.64	Protein DJ-1	Park7	1.78	Plasminogen activator inhibitor 1 RNA-binding protein	Serbp1
−0.68	Complement component 1 Q subcomponent-binding protein	C1qbp	1.58	RuvB-like 2	Ruvbl2
−0.74	Transcription factor BTF3	Btf3	1.57	Far upstream element-binding protein 1	Fubp1
−0.90	Peroxiredoxin-6	Prdx6	1.53	Aldehyde dehydrogenase, cytosolic 1	Aldh1a7
−0.95	Eukaryotic translation initiation factor 3 subunit F	Eif3f	1.43	Ribosome-binding protein 1	Rrbp1
−1.01	Proteasome activator complex subunit 1	Psme1	0.97	Lamin-A/C	Lmna
−1.02	Pulmonary surfactant-associated protein B	Sftpb	0.87	Phosphate carrier protein, mitochondrial	Slc25a3
−1.23	Platelet-activating factor acetylhydrolase IB subunit beta	Pafah1b2	0.86	Surfeit locus protein 4	Surf4
−1.30	GTP-binding nuclear protein Ran	Ran	0.54	Myosin-14	Myh14
−1.33	Phosphatidylethanolamine-binding protein 1	Pebp1	0.54	Ras-related C3 botulinum toxin substrate 1/3	Rac1/3
−1.34	6-phosphogluconolactonase	Pgls	0.38	Myosin-9	Myh9
−1.35	Splicing factor 1	Sf1	0.36	Complement C3	C3
−1.37	Macrophage migration inhibitoryv factor	Mif	−0.38	Poly(rC)-binding protein 2	Pcbp2
−1.37	Apolipoprotein A-IV	Apoa4	−0.43	Transketolase	Tkt
−1.42	Actin-related protein 2/3 complex subunit 4	Arpc4	−0.47	Transgelin-2	Tagln2
−1.45	Cystatin-B	Cstb	−0.49	Glutathione S-transferase P 1/2	Gstp1/2
−1.71	Peroxiredoxin-5	Prdx5	−0.51	Chloride intracellular channel protein 1	Clic1
−1.94	S100 calcium-binding protein A11	S100a11	−0.53	26S proteasome non-ATPase regulatory subunit 13	Psmd13
			−0.53	Peroxiredoxin-1	Prdx1
			−0.59	Tropomyosin alpha-4 chain	Tpm4
			−0.64	Protein DJ-1	Park7
			−0.68	Complement component 1 Q subcomponent-binding protein, mitochondrial	C1qbp
			−0.95	Eukaryotic translation initiation factor 3 subunit F	Eif3f
			−1.01	Proteasome activator complex subunit 1	Psme1
			−1.02	Pulmonary surfactant-associated protein B	Sftpb
			−1.23	Platelet-activating factor acetylhydrolase IB subunit beta	Pafah1b2
			−1.31	GTP-binding nuclear protein Ran	Ran
			−1.33	Phosphatidylethanolamine-binding protein 1; Hippocampal cholinergic neurostimulating peptide	Pebp1
			−1.34	6-phosphogluconolactonase	Pgls
			−1.37	Macrophage migration inhibitory factor	Mif
			−1.37	Apolipoprotein A-IV	Apoa4
			−1.42	Actin-related protein 2/3 complex subunit 4	Arpc4
			−1.45	Cystatin-B	Cstb
			−1.62	Zyxin	Zyx
			−1.68	2,4-dienoyl-CoA reductase, mitochondrial	Decr1
			−1.71	Peroxiredoxin-5	Prdx5
			−1.72	Transforming growth factor beta-1-induced transcript 1 protein	Tgfb1i1
			−1.94	S100 calcium-binding protein A11	S100a11
			−2.00	Transcription factor BTF3 homolog 4	Btf3l4

Differentially expressed proteins in lungs of P7 vitamin D deficient vs. replete mice using two analytical approaches; 1) based on the subset of proteins detected in all samples or, 2) based on proteins detected in a minimum of three biological replicates in one or more treatment groups where missing LFQ values were imputed to facilitate the statistical analysis

### Validation of protein expression

**Fig. 4:**
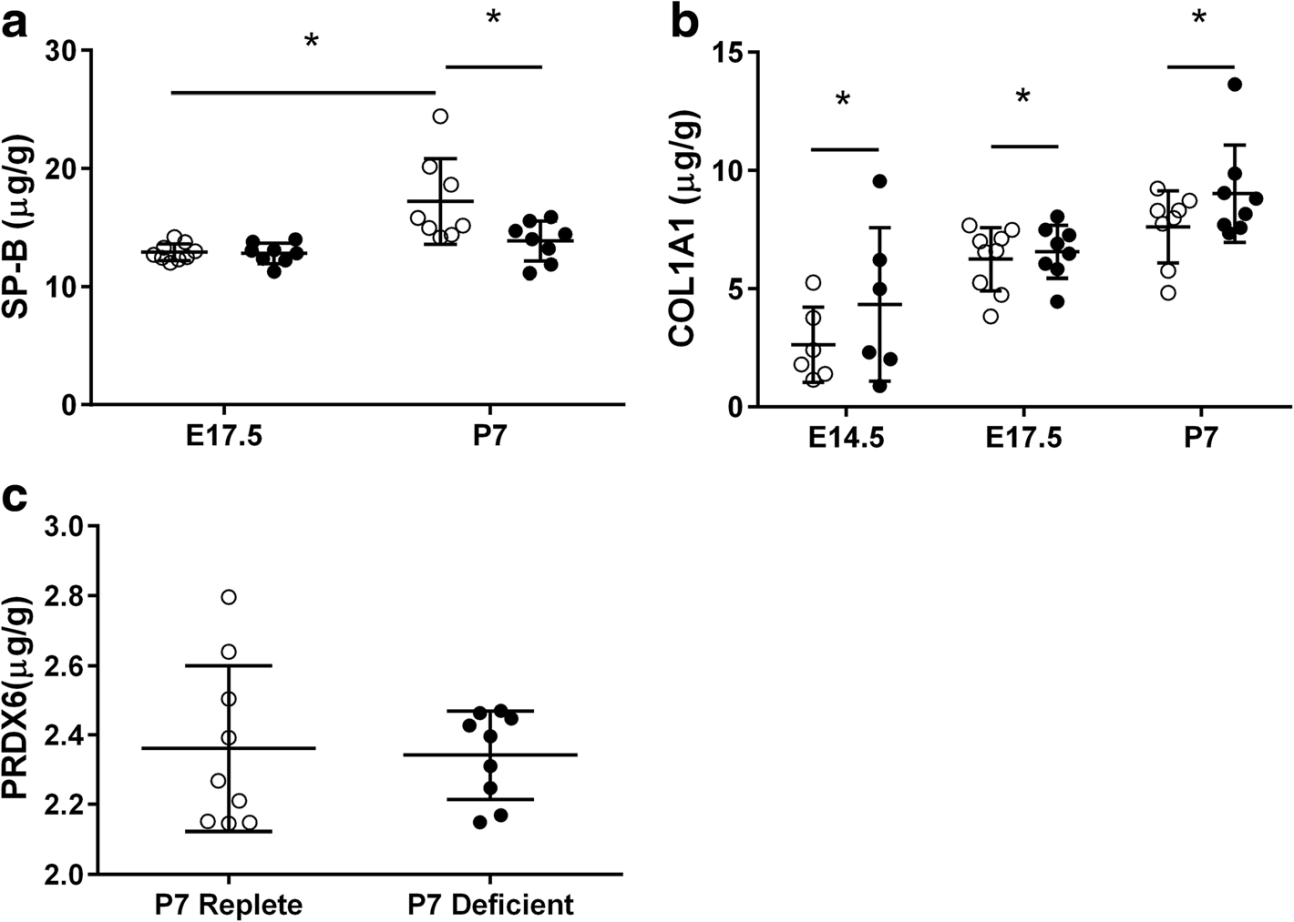
Protein validation of SP-B, COL1A1 and PRDX6. The expression of SP-B (**a**), COL1A1 (**b**) and PRDX6 (**c**) in the lungs of vitamin D replete (*white circles*) and deficient (*black circles*) mice assessed by ELISA. Data are represented as mean (SD), * indicates *p* < 0.05, *n* = 6/group for E14.5, *n* = 8-9/group for E17.5 and P7

**Fig. 5:**
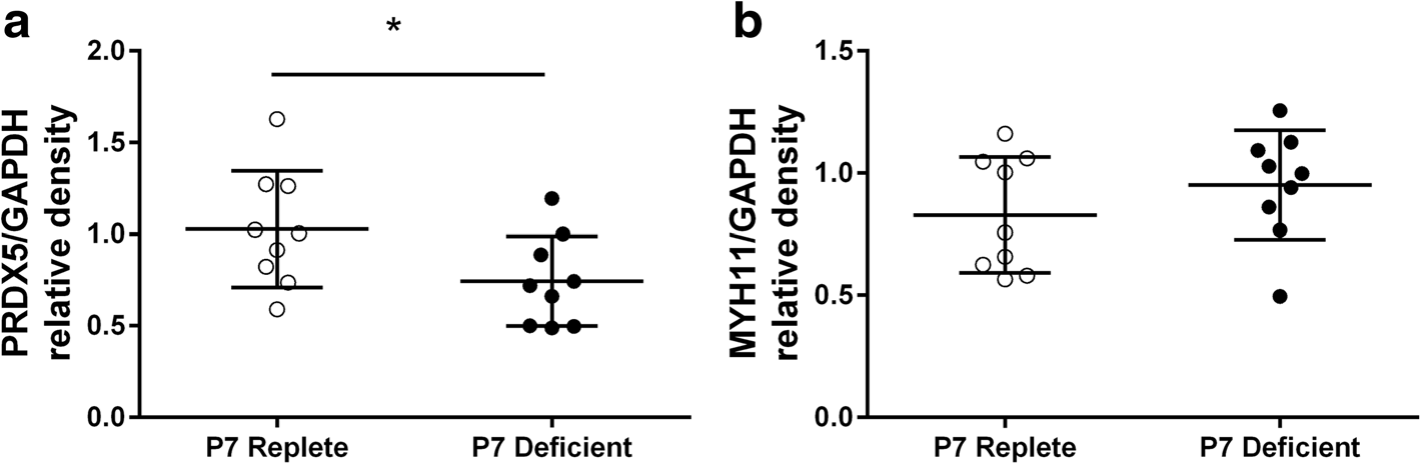
Protein validation of PRDX5 and MYH11. The relative density of PRDX5 (**a**) and MYH11 (**b**) in the lungs of vitamin D replete (*white circles*) and deficient (*black circles*) mice were assessed by western blot. Data are represented as mean (SD), * indicates *p* < 0.05, *n* = 9/group

Based on the proteins that were identified as differentially expressed at P7, we validated the expression of SP-B, PRDX6, and COL1A1 in the mice lungs using ELISA and the expression of PRDX5 and MYH11 using western blotting. SP-B was only measured in E17.5 and P7 mice because there are differentiated pulmonary epithelial cells capable of producing surfactant proteins by E17.5 (but not E14.5) [33]. There was a significant reduction in SP-B in lungs of vitamin D deficient mice compared to vitamin D replete mice at P7 (*n* = 8/group) (*p* = 0.002) but not at E17.5 (*n* = 8/group) (*p* = 0.90) (Fig. 4a). The expression of COL1A1 increased as the age increased (*p* < 0.001), and COL1A1 expression was higher in the lungs of vitamin D deficient mice compared to vitamin D replete mice (*n* = 6–9/group) across all three developmental time-points (*p* = 0.048) (Fig. 4b). There was no difference in PRDX6 expression in the P7 lungs between vitamin D replete and vitamin D deficient mice (*n* = 9/group) (*p* = 0.832) (Fig. 4c). However, the expression of PRDX5 was decreased in P7 lungs of vitamin D deficient mice compared to P7 lungs of vitamin D replete mice (*n* = 9/group) (*p* = 0.049) (Fig. 5a). The expression of MYH11 in P7 lungs was not different between vitamin D replete and vitamin D deficient mice (*n* = 9/group) (*p* = 0.274) (Fig. 5b).

### Lung structure

**Fig. 6:**
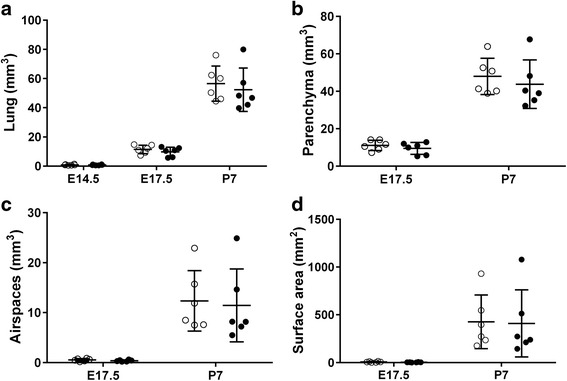
Lung morphometric parameters. Stereological estimation of lung volume (**a**), parenchymal volume (**b**), volume of airspaces in the parenchyma (**c**), and surface area of the airspaces (**d**) in the lungs of vitamin D replete (*white circles*) and deficient (*black circles*) mice. Data are represented as mean (SD), *n* = 6/group

E14.5 represents the pseudoglandular of lung development and as such many of the lung sub-structures are not fully developed [34]. Therefore, Vp, Va and Sa could not be assessed in the E14.5 lungs. There was no difference in V_L_ (*P* = 0.32) (Fig. 6a) between vitamin D deficient and replete mice (*n* = 6/group) at any age. At E17.5 and P7, there were no differences in Vp (*p* = 0.22) (Fig. 6b), Va (*p* = 0.29) (Fig. 6c) or Sa (*p* = 0.32) (Fig. 6d) between the lungs of vitamin D deficient and replete mice (*n* = 6/group).

## Discussion

E14.5, E17.5 and P7 are three key lung development time-points that represent the pseudoglandular, late canalicular/early saccular and alveolar development stages respectively [35]. This study has clearly shown that maternal vitamin D deficiency alters protein expression in the neonatal lung; particularly during the alveolarization stage of lung development. In addition, maternal vitamin D deficiency had no observable impact on lung morphology at these three time-points. The differential expression of proteins SP-B, COL1A1, and PRDX5 in the P7 vitamin D deficient lungs suggests that the expression of vitamin D sensitive proteins is not evident until later stages of neonatal lung development. Importantly, SP-B and COL1A1 have biologically plausible associations with lung structure and function [36, 37], which are consistent with the postnatal lung function changes associated with maternal vitamin D deficiency [17, 18]. The reduction in expression of PRDX5, a cytoprotective antioxidant enzyme [38], in the vitamin D deficient lung may impair defence against oxidative damage induced by secondary insults. Taken together, these data suggest that maternal vitamin D deficiency has the potential to impact lung development through reduced surfactant synthesis, increased collagen deposition, and impaired anti-oxidative stress response. The potential role of these proteins in the alteration of lung function is discussed below.

SP-B is a lipid-associated protein found in lung surfactant which is produced by type 2 alveolar cells and plays a critical role in the function of pulmonary surfactant [39]. SP-B rearranges lipid molecules to form a thin monolayer on the surface of the fluid lining at the gas-fluid interface which reduces the surface tension and promotes alveolar inflation [40]. Previous studies have shown that vitamin D stimulates phosphatidylcholine (PC) and phosphatidylglycerol (PG) biosynthesis and lamellar body release, which are associated with surfactant synthesis, in fetal rat lungs [25]. Our observations have shown decreased SP-B protein expression in the P7 lungs of vitamin D deficient mice. Collectively, these data suggest a critical role for vitamin D in facilitating normal surfactant function during lung development. Given the importance of surfactant in reducing compliance and maintaining airway patency [41] and the association between impaired surfactant function and respiratory disease [42], these observations highlight the importance of maintaining vitamin D levels during lung development. Impairments in surfactant synthesis are entirely consistent with the deficits in lung function that are associated with maternal vitamin D deficiency in animal [25] and human studies [43]. Previous studies using mouse models have shown that one of the key effects of early life vitamin D deficiency is a reduction in lung volume and increased lung stiffness in P14 mice [17]. In a retrospective analysis of a longitudinal birth cohort we also demonstrated that maternal vitamin D deficiency at 16–18 weeks gestation was associated with a reduction in forced expiratory volume (FVC) [13]. One possible explanation for these observations is a reduction in pulmonary surfactant, which is consistent with data from the present study. Interestingly, in previous birth cohort study, maternal vitamin D deficiency was measured at the canalicular stage of human lung development while in the present study we found that vitamin D deficiency was primarily associated with changes in protein expression at early alveolar stages of mouse lung development. This may suggest that the sensitivity of the lung to vitamin D deficiency varies between mice and humans; although we only measured vitamin D levels at one time-point during gestation in the human study and it is entirely possible that the mothers were deficient throughout gestation. While reduced surfactant production is one explanation for the reduction in lung volume and decreased compliance in early postnatal lung development [17], it could also be explained by changes in tissue structural proteins, such as collagen, which is other major observation in our study.

Collagens are present in major structures of the lung, and any alterations in quantity, structure, or geometry of their distribution would be likely to alter the lung function. Particularly, changes in the interstitium would have dramatic effects on lung function, where the distance between air and blood may be as little as 50 nm [44]. COL1A1 encodes collagen type Ι which is a major structural protein in the lung interstitium and is produced in large quantities during lung development and during pathological fibrotic processes [45]. In vitro studies have shown that the addition of vitamin D reduces the expression of collagen type Ι [46], and induces an anti-fibrotic phenotype in various types of cells [23]. In our study, COL1A1 was increased in lungs of vitamin D deficient mice suggesting that collagen synthesis during lung development is also sensitive to vitamin D. Again, this is consistent with the functional defects associated with maternal vitamin D deficiency that have been described previously [17, 18]. It was interesting to note that differences in COL1A1 were only evident at the P7 time-point from the LC-MS/MS assay, whereas the ELISA analysis showed that vitamin D deficiency increased the expression of COL1A1 at E14.5, E17.5 and P7. Notwithstanding this discrepancy, our data suggest that vitamin D deficiency increases the synthesis of collagen type Ι during early lung development.

Using two differential analysis approaches to analyse the LC-MS/MS assay data we have identified differential expression of a range of proteins including peroxidredoxin (PRDX) 1, 5, and 6, which were decreased in P7 lungs of vitamin D deficient mice compared to vitamin D replete mice and myosin (MYH) 9, 11, and 14 were increased in P7 lungs with vitamin D deficiency. While we were unable to validate the differential expression of PRDX6 of MYH11 in independent samples, we are able to confirm that the expression of PRDX5 was reduced in the lungs of vitamin D deficient mice at P7. PRDXs are a family of peroxidases which are present in aerobic organisms and have an important role in protection of human tissue from endogenous and exogenous oxidative damage [47]. PRDXs are classified into typical 2-Cys (PRDX1-4), atypical 2-Cys (PPXD5), and 1-Cys (PRDX6) PRDX subfamilies. These enzymes degrade hydrogen peroxide (H_2_O_2_) using their thiol groups of cysteines (Cys) as catalytic centres [47, 48]. Various studies have shown the overexpression of PRDXs providing significant protection against hyperoxia and apoptosis, and the deficiency of these enzymes can enhance lipid peroxidation [49–52]. PRDX1, 3, 5, and 6 have prominent and cell specific expression in human lung tissue [53, 54]. PRDX5 plays a protective role in against oxidative stress by reducing apoptosis in human tendon cells and lung carcinoma cells [51, 55]. Furthermore, studies have indicated that PRDX5 has function in maintaining collagen synthesis and protecting against fibrosis [51, 56], which is consistent with our finding that overexpression of collagen in lungs of maternal vitamin D deficient mice in early life may be one of the driven factors of impaired lung function in later life.

We were unable to confirm the differential expression of smooth muscle heavy chain MYH11I which we identified by LC-MS/MS. Given that we did not independently quantify MYH4 and 9, it is possible that these proteins do play a role in vitamin D deficiency induced alterations in lung function. In particular, MYH has been linked to airway smooth muscle contractility [57] which is consistent with our previous observation that maternal vitamin D deficiency causes increased responsiveness of the airways to bronchoconstricting agents [18]. The potential role of MYH in vitamin D deficiency induced impairments in lung function requires further study.

The lack of difference in lung morphology between vitamin D deficient and replete mice across these three developmental time-points that we have shown in this study suggests that vitamin D deficiency has little impact on gross lung structure during the early stages of lung development. In contrast, our previous findings have shown that lung volume is reduced in P14 mice that were vitamin D deficient *in utero* [17] while parenchymal volume and the volume of the air in the alveolar ducts are reduced in adult vitamin D deficient mice [18]. This is, however, consistent with the observation that differences in protein expression were not evident until later in lung development and it may be that these differences in protein expression take time to manifest into structural differences at the whole organ level.

This study had some limitations that should be acknowledged. Firstly, as with any complex tissue, the depth of proteome coverage is limited to the most abundant proteins. However, when working with small quantities of embryonic tissue there are practical limitations to sample fractionation for lower abundance protein detection. Secondly, because we used whole lung homogenates we are unable to comment on the cell specific differences in protein expression. Thirdly, in this study we only used lungs from female offspring due to the fact that females are more sensitive to the effect of vitamin D deficiency on lung development [17, 58]. Thus, it is unclear whether the same effects of vitamin D deficiency on protein expression are evident in male lungs.

## Conclusions

In summary, the lack of difference in protein expression in prenatal developmental time-points, but differential protein expression in the early postnatal time-point suggests that vitamin D deficiency may induce alterations in lung function during alveolarization by altered surfactant and collagen synthesis. The lack of difference in lung structure at P7, in contrast to structural alterations at P14 [17], suggests that the effect of early life vitamin D deficiency on lung structure is not realised until later in alveolar development. These data provided a plausible mechanism linking maternal vitamin D deficiency with altered postnatal lung function and highlight the importance of vitamin D level during normal lung development.
